# A New Regression Model Based on Salivary Biomarkers, Chronological Age and Gender to Predict the Stage of Cervical Vertebral Maturation in Orthodontic Patients 

**DOI:** 10.30476/dentjods.2025.103220.2431

**Published:** 2025-09-01

**Authors:** Asma Sookhakian, Maryam Zahed, Hamidreza Pakshir, Shabnam Ajami, Maryam Zangooi Booshehry

**Affiliations:** 1 Postgraduate Student, Student Research Committee, School of Dentistry, Shiraz University of Medical Sciences, Shiraz, Iran.; 2 Oral and Dental Disease Research Center, Dept. of Oral and Maxillofacial Medicine, School of Dentistry, Shiraz University of Medical Sciences, Shiraz, Iran.; 3 Orthodontics Research Center, School of Dentistry, Shiraz University of Medical Sciences, Shiraz, Iran.; 4 Oral and Maxillofacial Radiologist, Private Practice, Shiraz, Iran.

**Keywords:** Alkaline Phosphatase, Biological models, Growth, Insulin-Like Growth Factor I, Orthodontics

## Abstract

**Background:**

In orthodontics, radiography-based methods are frequently used for the assessment of skeletal maturity. Concerning X-ray exposure hazard, a new objective and less invasive method is needed to identify the optimal orthodontic treatment timing.

**Purpose:**

In this study, the pattern of salivary alkaline phosphatase and insulin-like growth factor-1 in the circumpubertal period was investigated. Moreover, new regression models were built to predict skeletal maturity more accurately.

**Materials and Method:**

In this cross-sectional study, fifty-five subjects aged 7 to 20 years were enrolled to compare the levels of salivary alkaline phosphatase and insulin-like growth factor-1 at different skeletal maturity stages by using 6-stage cervical vertebral maturation method. Moreover, three new multinomial logistic regression models based on these biomarkers, as well as chronological age, and gender were built to predict the cervical stage. Statistical analysis was performed using SPSS (version 24) software. In addition to descriptive statistics, the Shapiro-Wilk test, the one-way analysis of variance test, the independent Samples T-test, the Pearson correlation coefficient, the Chi-square test, and the overall correct classification rate was performed.

**Results:**

A significant difference was observed for salivary alkaline phosphatase and also insulin-like growth factor-1 levels between cervical stages (< 0.001). The peak level in both salivary alkaline phosphatase and insulin-like growth factor-1 levels was observed at CS3. The levels of these biomarkers had a significant positive correlation with the cervical stages from CS1 to CS3 and a significant negative correlation from CS3 to CS6. Based on the regression model analysis, we found that Model 3´ which combined gender with chronological age, alkaline phosphatase, and insulin-like growth factor-1 had the highest predictive ability (76.4%).

**Conclusion:**

The notably larger predictive ability of the new model which combined gender with chronological age, salivary alkaline phosphatase, and salivary insulin-like growth factor-1 might be responsible for the identification of the optimal orthodontic treatment timing in an objective and less invasive manner in healthy growing individuals.

## Introduction

The timing of the orthodontic and dentofacial orthopedic treatment onset may be as critical as the treatment planning [ 1 - 3 ]. One of the most important objectives of orthodontic treatments is to treat skeletal discrepancies using growth potential [ 4 ]. Therefore, the correct identification of growth phases through the skeletal maturity assessment is important in diagnosis and treatment planning [ 5 - 6 ]. 

Individual skeletal maturity is frequently assessed using hand-wrist analysis and the cervical vertebral maturation (CVM) methods in orthodontics [ 7 ]. However, nowadays newer possibilities are offered by biomarkers that are involved in bone remodeling and growth [ 8 ]. Alkaline phosphatase (ALP) and insulin like growth factor-1 (IGF-1) are two certain examples of such biomarkers. On the other hand, biomarkers can be measured in different biologic fluids. Recently, interest in salivary diagnostics has advanced in medicine and dentistry . 

ALP is a membrane-bound enzyme that hydrolyses monophosphate ester bonds and increases the local phosphate ion concentration [ 11 ]. It is also one of the primary biomarkers in the osteogenesis process [ 12 ]. Therefore, ALP level has been proposed as an indicator for growth phase identification in several studies . 

IGF-1 mediates growth hormone to promote musculoskeletal growth [ 16 ]. In addition to identifying growth disorders, IGF-1 can also predict the skeletal maturity in order to attain favorable results in orthodontic treatments [ 17 ]. Many studies have been conducted correlating serum IGF-1 levels with different skeletal maturity stages and proposed the role of serum IGF-1 as a skeletal maturity indicator [ 18 - 20 ]. However, blood sampling by intravenous method remains an invasive procedure and can cause discomfort for the patients [ 21 ].

A study using the CVM method to evaluate the best timing for dentofacial orthopedic treatments emphasized that protocols designed to promote or limit lower jaw growth are most effective when applied during the circumpubertal phase, which coincides with the growth spurt. For instance, deficiencies in mandibular ramus height and increased facial height can be significantly improved if treatment occurs at the peak of mandibular growth (CS3). Conversely, protocols targeting maxillary changes, such as protraction or expansion, yield better results when applied during the pre-pubertal stage (CS1 or CS2) [ 1 ]. However, CVM method like other radiography-based methods has X-ray exposure hazard. Therefore, a new less invasive and objective method compared to radiography is needed to identify the optimal orthodontic treatment timing. Despite the advantages of salivary biomarkers such as easy collection, smaller sample volume, favorable patient compliance, higher sensitivity and correlation with blood levels [ 22 ], a few studies have investigated salivary biomarkers in relation to skeletal maturity [ 22 - 23 ]. A recent systematic review was conducted by Khade *et al*. [ 23 ] to assess the reliability of salivary biomarkers as skeletal maturity indicators in 2023. Based on their study findings, salivary biomarkers can be used as an adjunct to other reliable methods for skeletal maturity assessment. However, there is a need for further research in this field. On the other hand, our previous cross-sectional study on skeletal maturity prediction yielded encouraging results [ 22 ]. Our findings indicated that using a ternary combination of salivary ALP and IGF-1 with chronological age (CA) can be verified as a less invasive method for skeletal maturity assessment [ 22 ]. In light of the above, further investigation is essential. Therefore, the aims of the present study were investigating the pattern of these salivary biomarkers in the circumpubertal period and establishing new regression models by incorporating gender along with CA, salivary ALP, and IGF-1 levels to predict the stage of CVM more accurately and subsequently to identify the optimal orthodontic treatment timing in growing healthy individuals. To the best of our knowledge, the present study is the first that addressed the question of whether a model including gender performs better than a general model for cervical stage prediction in orthodontic patients. 

## Materials and Method

### Study Population

This cross-sectional study was carried out on subjects who were either starting orthodontic treatment or already undergoing orthodontic treatment at Shiraz School of Dentistry in Iran. Data from the subjects were taken from the complete records of our previous study on skeletal maturity assessment [ 22 ]. The study protocol was according to the ethical standards of the institutional research ethical committee (Ethical code: IR.SUMS. DENTAL.REC.1399.197). It is important to note that a written informed consent was obtained from all subjects. For the participants under 18 years, the legal guardians of the child signed the consent form.

This study included healthy males and females aged 7 to 20 years with confirmed birth records and a recent lateral cephalogram (taken within the past 6 months) showing clear images of the second, third, and fourth cervical vertebrae for orthodontic evaluation.

Any subject diagnosed with systemic disease (based on medical history, signs, and symptoms), growth abnormalities, nutritional problems (based on height, weight, and body mass index charts), self-reported pregnancy or lactation, history of injury or surgical intervention in the cervical spine, history of xerostomia, and taking medications such as vitamin preparations, calcium supplements, anti-inflammatories or antibiotics in the last month prior to study enrollment were excluded [ 22 ].

### Clinical Monitoring and Saliva Collection Procedure

Saliva samples were collected from all participants during the same season (spring 2021) in the morning to avoid circadian changes in salivary flow. They were instructed to avoid drinking, eating, chewing gum, and brushing their teeth for at least 90 minutes prior to collecting the sample. Before sample collection, participants rinsed their mouths with water and rested for five minutes. Then, they sat comfortably and 3 ml unstimulated whole saliva was collected using spitting method [ 22 ]. Immediately after saliva collection, samples were kept in a thermal box with ice packs (maintained at temperature 2-8°) and sent to the biochemistry laboratory.

### Biochemical Analysis of Salivary Samples

The saliva samples were spun at 3000rpm for 10 minutes to remove impurities and oral cells, reducing turbidity. The clear liquid (supernatant) was divided into two parts. One part was analyzed immediately to measure baseline ALP levels, as these levels decrease significantly over time at -20°C. The other part was frozen at -20°C until used to measure IGF-1 levels. A single operator, unaware of the patients’ age and puberty status, conducted the biochemical tests. To reduce bias, measurements were repeated, and the average values were reported.

The ALP level was determined quantitatively based on the DGKC (the Standard of German Society for Biochemistry) using a Human ALP Kit (Biorexfars, Iran) and an auto analyzer at a wavelength of 405 nm, according to the manufacturer’s instructions. The level of ALP was reported in Units per Liter (U/L). Additionally, the IGF-1 level was quantitatively determined by Enzyme-Linked Immunosorbent Assay (ELISA) using Human IGF-1 ELISA Kit (ZellBio GmbH, Germany), according to the manufacturer’s instructions. The optical density was measured using a micro plate reader at a wavelength of 450 nm. Finally, the IGF-1 level was reported in nanograms per milliliter (ng/ml). 

### Individual Skeletal Maturity Assessment

All the subjects were grouped to six cervical stages (CS1 to CS6) according to the Baccetti’s CVM method [ 1 ]. This method assessed the presence or absence of concaveness on the lower border of the C2, C3, and C4 vertebral bodies, as well as morphologic form of the C3 and C4 vertebral bodies (trapezoid, rectangular horizontal, square, and rectangular vertical). It should be noted that all the lateral cephalograms were evaluated by two blinded examiners. One additional blinded examiner helped as a reference examiner in cases of disagreement. 

### Statistical Analysis

Statistical analysis was conducted on the data obtained from our previous records using SPSS (version 24) software. This study used a dummy variable for gender to differentiate between male and female participants. The chronological age (CA) and salivary levels of ALP and IGF-1 were measured as quantitative variables. In addition to descriptive statistics, the following statistical analysis was done. The Chi-square test was used to analyze the sample distribution of different cervical stages and balancing of the groups by gender. The Shapiro-Wilk test evaluated the normality of the distribution of the variables. Since data followed normal distribution, parametric tests were used: The one-way analysis of variance (ANOVA) test was used to compare the mean ALP and IGF-1 levels corresponding to the six cervical stages. If the p Value (P) in ANOVA was less than 0.05, a Post Hoc test (Bonferroni multiple comparison) was performed to detect differences between the two stages. The mean ALP and IGF-1 levels between the genders in each cervical stage were compared using the independent samples T-test. The Pearson correlation coefficient was calculated between the levels of ALP and IGF-1 and the cervical stages.

Three new multinomial logistic regression models were used to predict the skeletal maturity based on the salivary ALP level, salivary IGF-1 level, CA, and gender. In the present study, a model was denoted by its predictors: (CA+ALP), for example, having CA and ALP effects. The adequacy of fit for a logistic regression model was assessed using the chi-square test of the mo-del coefficients. The models’ predictive accuracy was evaluated using McFadden’s pseudo R^2^ (R^2^_MF_) to determine the overall correct classification rate. All analyses were considered statistically significant at *p*< 0.05.

In the present study, Logit models pair each response category with the last one (CS6) as the baseline category: $\begin{array}{cc} \mathrm{Log}\frac{\pi_{j}(x)}{\pi_{J}(x)}=\alpha_{j}+{\beta \prime }_{j}xj=1,\mathrm{...},J-1 & \left(A\right) \end{array}$

These J – 1 equations determine parameters for logits with other pairs of response categories, since 

$\begin{array}{cc} \frac{\pi_{a}(x)}{\pi_{b}(x)}=\frac{\pi_{a}(x)}{\pi_{J}(x)}-\frac{\pi_{b}(x)}{\pi_{J}(x)} & \left(B\right) \end{array}$

The equation that expresses multinomial logit models directly in terms of response probabilities $\left\{\pi_{j}(x)\right\}$ is

$\begin{array}{cc} \pi_{j}(x)=\frac{\exp(\alpha_{j}+{\beta \prime }_{j}x)}{1+\sum_{\mathrm{h=1}}^{\mathrm{J-1}}\exp(\alpha_{h}+{\beta \prime }_{h}x)} & \left(C\right) \end{array}$

For the baseline category (CS6), αJ and βJ should be equal to zero to have identifiable reasons. The denominator of (C) is the same for each j. The numerators for various j sum to the denominator, so $\sum_{j}\pi_{j}(x)=1$ [ 24 ]. 

## Results

Fifty five healthy subjects (both females and males) aged 7 to 20 years were enrolled. Sample distribution among the cervical stages and sex distribution in each stage was statistically uniform (*p*> 0.05). 

**Figure 1:**
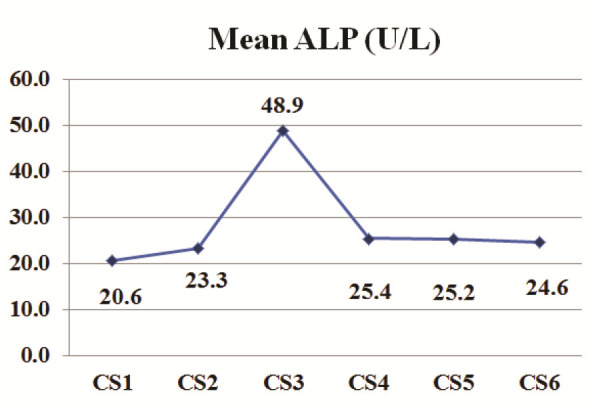
ALP pattern in the circumpubertal period, ALP: alkaline phosphatase, CS1-CS6: cervical stage 1 through cervical stage 6, U/L: Units per Liter

**Figure 2:**
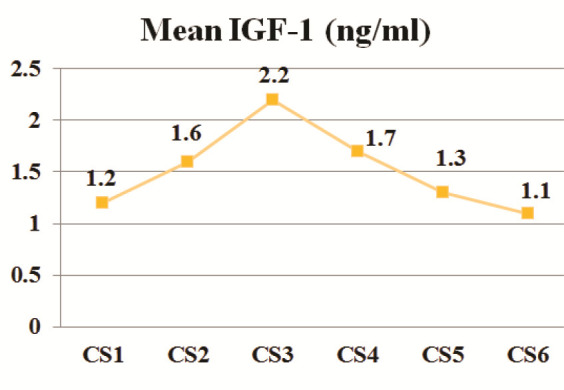
IGF-1 pattern in the circumpubertal period, CS1- CS6: cervical stage 1 through cervical stage 6, IGF-1: insulin like growth factor-1, ng/ml: nanograms per milliliter

The descriptive analysis of salivary ALP and IGF-1 levels for each cervical stage is shown in Table 1 and presented graphically in Figures 1-2. The one-way ANOVA test revealed a significant difference in means for salivary ALP and IGF-1 between the cervical stages (*p*< 0.001). Table 2 lists the intergroup comparison of these biomarkers between different cervical stages using the Bonferroni test. The result of this test showed that the salivary ALP level at CS3 was significantly higher than salivary ALP levels at CS1, CS2, CS4, CS5, and CS6. In addition, the salivary IGF-1 level at CS3 was significantly higher than CS1, CS2, CS5, and CS6; whereas no significant difference was found between CS3 and CS4. On further analysis, the salivary ALP and IGF-1 levels showed no significant differences between the genders within each cervical stage (*p*> 0.05).

**Table 1:** Descriptive statistics of salivary ALP and IGF-1

Cervical stage	N	Salivary ALP (U/L)			Salivary IGF-1 (ng/ml)		
		Mean±SD	Min-Max	95% CI for Mean (LB-UB)	Mean±SD	Min-Max	95% CI for Mean (LB-UB)
1	9	20.57±5.79	11.00-28.60	16.12 –25.02	1.23±0.23	0.88–1.62	1.05 – 1.41
2	11	23.25±10.82	9.35 –48.00	15.98 – 30.52	1.57±0.52	0.83-2.54	1.22 – 1.92
3	9	48.85±19.10	17.90-74.00	34.17 – 63.53	2.23±0.68	1.35–3.02	1.70 – 2.75
4	6	25.37±7.21	12.20-3s2.00	17.80 – 32.93	1.71±0.62	1.01–2.51	1.06 – 2.36
5	9	25.18±13.43	11.00-53.00	14.86 – 35.51	1.25±0.42	0.65–1.88	0.93 – 1.57
6	11	24.56±5.59	12.50-32.00	20.81 – 28.32	1.10±0.20	0.68–1.35	0.97 – 1.24

ALP: Alkaline phosphatase, CA: Chronological age, CI: Confidence interval, IGF-1: Insulin like growth factor-1, LB: lower bond, Max: Maximum, Min: Minimum , N: Number of subjects in each stage, ng/ml: Nanograms per milliliter, SD: Standard deviation, UB: Upper bond, U/L: Units per liter

**Table 2:** Bonferroni multiple comparison for mean salivary levels of ALP and IGF-1 at different cervical stages

Bonferroni multiple comparison	Salivary ALP (U/L)		Salivary IGF-1 (ng/ml)	
	Mean difference between two groups	p Value	Mean difference between two groups	p Value
CS1 vs. CS2	-2.68	1.000	-0.34	1.000
CS1 vs. CS3	-28.28	0.000*	-1.00	0.001*
CS1 vs. CS4	-4.80	1.000	-0.48	0.815
CS1 vs. CS5	-4.62	1.000	-0.02	1.000
CS1 vs. CS6	-4.00	1.000	0.13	1.000
CS2 vs. CS3	-25.60	0.000*	-0.65	0.044*
CS2 vs. CS4	-2.12	1.000	-0.14	1.000
CS2 vs. CS5	-1.93	1.000	0.32	1.000
CS2 vs. CS6	-1.31	1.000	0.47	0.335
CS3 vs. CS4	23.48	0.004*	0.51	0.618
CS3 vs. CS5	23.67	0.001*	0.97	0.001*
CS3 vs. CS6	24.29	0.000*	1.12	0.000*
CS4 vs. CS5	0.18	1.000	0.46	0.984
CS4 vs. CS6	0.80	1.000	0.61	0.195
CS5 vs. CS6	0.62	1.000	0.15	1.000

ALP: Alkaline phosphatase, CS1-CS6: Cervical stage 1 through cervical stage 6, IGF-1: insulin like growth factor-1, ng/ml : nanograms per milliliter, U/L: units per liter * *p* Value< 0.05 considered significant

The correlation coefficient between the salivary ALP level and the cervical stages was positive from CS1 to CS3 (r= +0.638, *p*< 0.001) and negative from CS3 to CS6 (r= -0.550, *p*= 0.001). Similarly, the Pearson correlation between the salivary IGF-1 level and the cervical stages from CS1 to CS3 showed a positive correlation coefficient (r= +0.623, *p*< 0.001), whereas the correlation coefficient from CS3 to CS6 was negative (r= -0.685, *p*< 0.001).

Table 3 contains the models´ fit statistics to compare their abilities for cervical stage prediction. The explanatory variables in all the models were statistically significant (*p*< 0.05). Upon comparing models including gender and general models, we found that combining gender as an explanatory variable to the models can improve model´s predictive ability. It is important to note that Model 3´ which combined gender with CA, salivary ALP, and salivary IGF-1 had the highest predictive ability. Results of this regression model (Model 3´) are summarized in Table 4. In the multivariable equations generated from Table 4, Gender =1 was defined for female and Gender =0 was defined for male. 

**Table 3:** Fit statistics of multinomial logit models for cervical stage prediction

Model	Likelihood-Ratio Chi-square Statistic (p Value)	df	R^2^MF	Correct Classification Rate (%)
Model 1: (CA+IGF-1)	91.958 (< 0.001)	10	0.471	58.2
Model 1´: (CA+IGF-1+Gender)	114.670 (< 0.001)	15	0.588	61.8
Model 2: (CA+ALP)	91.784 (< 0.001)	10	0.470	65.5
Model 2´: (CA+ALP+Gender)	118.325 (< 0.001)	15	0.606	70.9
Model 3: (CA+ALP+ IGF-1)	107.802 (< 0.001)	15	0.552	70.9
Model 3´: (CA+ALP+IGF-1+Gender)	129.491 (< 0.001)	20	0.664	76.4

Alkaline phosphatase (ALP), chronological age (CA), insulin like growth factor-1 (IGF-1), mcFadden´s pseudo R^2^ (R^2^MF)

**Table 4:** Estimated parameters in Model 3´ for cervical stage prediction

Logit	j	Intercept (α_j_)	(Β_j_)			
			CA	ALP	IGF-1	Gender
Log (π_CS1_/π_CS6_)	1	145.766	-9.404	0.026	-8.928	-13.372
Log (π_CS2_/π_CS6_)	2	130.143	-8.227	0.021	-7.015	-10.683
Log (π_CS3_/π_CS6_)	3	90.917	-5.978	0.167	-3.821	-6.958
Log (π_CS4_/π_CS6_)	4	98.987	-5.803	0.034	-6.603	-7.324
Log (π_CS5_/π_CS6_)	5	81.751	-4.321	0.003	-8.030	-5.561

ALP: Alkaline phosphatase, CA: chronological age, IGF-1: Insulin like growth factor-1

## Discussion

This cross-sectional study aimed to evaluate the use of salivary biomarkers ALP and IGF-1 as indicators of skeletal maturity for orthodontic clinical assessment. Additionally, this study introduced three new multinomial logistic regression models to investigate whether a model that includes gender outperforms a general model for assessing skeletal maturity. 

We observed that the salivary ALP and IGF-1 levels gradually rise from CS1 to CS3 (Peak level: ALP = 48.9 U/L and IGF-1= 2.2 ng/ml), followed by a decline up to CS6. In further detail, significant increases in salivary ALP and IGF-1 levels were observed at CS3 compared to all other cervical stages. The only exception was a minimal and insignificant difference in IGF-1 levels between CS3 and CS4. According to Perinetti *et al*. [ 6 ], CS3 can also be described as “during pubertal” growth phase. In fact, they categorized the growth phases as pre-pubertal (CS1 and CS2), pubertal (CS3 and CS4), and post-pubertal (CS5 and CS6). Therefore, we refer to CS3 and CS4 as “during pubertal” to permit equivalence of comparison with other studies. Moreover, there were no significant differences in the biomarker levels between the two pre-pubertal (CS1 and CS2) and the two post-pubertal (CS5 and CS6) stages. This could be due to the fact that the levels of these biomarkers decline to the pre-pubertal levels after puberty. The results also showed that the salivary ALP and IGF-1 were statistically significant in cervical stage prediction. Based on these outcomes, we recommend that ALP and IGF-1 salivary biomarkers be viewed as dependable markers of skeletal maturity. 

Currently, radiographs are commonly used to predict the pubertal growth phase and estimate the remaining growth potential by observing the organized ossification of specific bones radiologically [ 22 , 25 ]. Interests in the maturational changes in the size and shape of cervical vertebrae has led to the development of various CVM methods, along with several modifications and improvements over time [ 26 - 27 ]. However, several limitations have been observed in CVM staging, such as the subjective disagreement and the need for X-ray exposure. Hence, a superior and less invasive method is needed to assess skeletal maturity. Nowadays, the advantages of salivary sampling make salivary biomarkers very attractive. ALP and IGF-1 are salivary biomarkers known to have variable levels during growth and maturation [ 15 ]. Normally, 50% of adults’ total serum ALP originates from the liver and the rest from bone, whereas 90% of the ALP activity is specific to bone in children and adolescents [ 28 ]. 

It has been proposed that IGF-1 stimulates growth systemically (as an endocrine hormone) as well as locally (as a paracrine and autocrine growth factor) [ 20 , 29 ]. In particular, this growth factor accelerates the growth, differentiation, and activity of substrate synthesis in osteoblasts and chondroblasts . Therefore, IGF-1 is crucial for both local and systemic regulation of longitudinal bone growth [ 30 ].

Regarding salivary ALP level, similar results were found by Tarvade *et al*. [ 9 ] and Hegde *et al*. [ 14 ]. They reported that the salivary ALP level reaches its highest point during the peak of pubertal growth in both genders, as determined by skeletal maturity assessments using MP3 in hand-wrist radiographs. However, Alhazmi *et al*. [ 31 ] reported that the peak level of the salivary ALP is during pre-pubertal period (at CS1). The difference reported can be attributed to the differential research methodology associated with the biochemical analysis. In fact, contrary to what they had done, ALP level was not normalized to total protein concentration in the saliva in the current samples as well as the previously referenced studies . Our findings also in line with two previous studies that have evaluated the GCF ALP level in relation to CVM stages . Perinetti *et al*. [ 6 ] found a twofold peak in GCF ALP level during the pubertal growth phase. The other study showed that the total GCF ALP level is more sensitive to the growth phase rather than normalized GCF ALP level and it was significantly greater in the pubertal growth phase [ 13 ].

With respect to IGF-1, the findings of the current study are consistent with those of a previous study by Ryan *et al*. [ 32 ] who determined the changing pattern of salivary IGF-1 level with age and revealed that the peak was during puberty. A previous study on IGF-1 level in relation to skeletal maturity using hand-wrist radiographs reported that the peak of serum IGF-1 level was significantly higher during pubertal phase [ 19 ]. Our findings also agreed with several studies on IGF-1, which examined the association between the serum IGF-1 level and CVM staging using different methods and reported a peak in serum IGF-1 level during the pubertal growth phase (at CS3 or CS4 of CVM staging) . Although Masoud *et al*. [ 30 ] reported that the peak of blood spot IGF-1 level was at CS5, there is a considerable consensus among the existing studies that the level of this biomarker peaks in the pubertal phase . 

Upon further examination, there were no notable differences observed between the female and male groups within each cervical stage in terms of salivary ALP and IGF-1 levels. This finding agreed with Ryan *et al*. [ 32 ] who also reported that salivary IGF-1 levels were similar in both genders. However, it is in contrast with the findings of the study conducted by Alhazmi *et al*. [ 31 ] that showed a significant difference in salivary ALP level between females and males.

Furthermore, in our recently published paper, we introduced a model using the combination of salivary ALP and IGF-1 with CA to determine the cervical stage with high predictive ability, which was 70.9% [ 22 ]. Until now, this model seems to be the best model presented in literature for correct prediction of cervical stage. However, the present study results showed that combining gender with CA, salivary ALP, and salivary IGF-1 provided a higher value for predictive ability, which reached 76.4%. In other words, we increased the amount of predictive ability from 70.9% to 76.4% with the addition of gender as an accessible and inexpensive variable (Table 3). This model enables a more precise assessment, and the multivariable equations derived from Table 4 can be used with basic computing software to clinically predict the cervical stage in healthy growing individuals. On the other hand, no published studies to the best of our knowledge have investigated the comparison between the abilities of models including gender and general models for cervical stage prediction. In this regard, three new multinomial logistic regression models were built to compare their overall results with those of their general models. Our findings indicated that combining gender as an explanatory variable to the models can improve the model´s predictive ability (Table 3). The explanation for this result may be the age advancement of females compared to males in skeletal maturation [ 22 , 33 - 34 ]. 

In this study, saliva collection was not conducted simultaneously with radiographic imaging due to ethical concerns. However, participants were included only if they had lateral cephalograms taken within the past six months, consistent with the methodology used in a prior study [ 31 ]. Additionally, we encountered a challenge to increase the sample size because the COVID-19 pandemic made the subjects less willing to participate in a clinical study. Our research aims to provide a basis for future longitudinal studies to determine diagnostic reference ranges for these biomarkers based on the stages of skeletal maturity. This will assist orthodontic practitioners in managing growth patterns during both ascending and descending phases. Moreover, future studies on larger populations are suggested for examining more variables that can affect skeletal maturity assessment. 

## Conclusion

The results of this study showed that the salivary ALP and IGF-1 levels appear to be less invasive indicators of skeletal maturity prediction. They have diagnostic potentials for the identification of the optimal timing in growing healthy individuals scheduled for orthodontic treatment. In addition, the model designed in this study that integrated gender with CA, salivary ALP, and salivary IGF-1 demonstrated superior predictive capability compared to models lacking gender and can be utilized for assessing the cervical stages in healthy growing individuals.
